# Supplementary material for: Splice-Junction-Based Mapping of Alternative Isoforms in the Human Proteome
Source: Cell Rep. Author manuscript; Available in PMC 2020 Jan 15. (PMC6961840; doi:10.1016/j.celrep.2019.11.026)
Supplement: 2 [file NIHMS1546469-supplement-2.zip › DF1/PXD009737/TestisLysC/TestisLysC_7_KTN1_NNSYGEWLHGFEK.pdf]

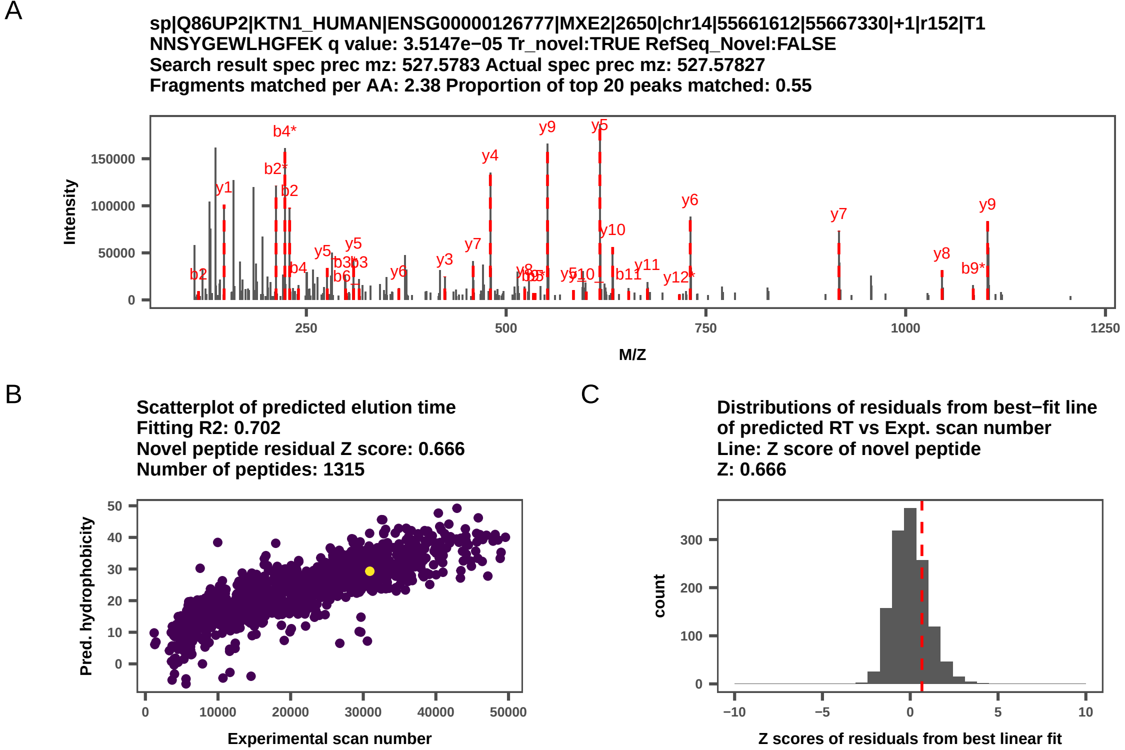

A
sp|Q86UP2|KTN1_HUMAN|ENSG00000126777|MXE2|2650|chr14|55661612|55667330|+1|r152|T1
NNSYGEWLHGFEK q value: 3.5147e−05 Tr_novel:TRUE RefSeq_Novel:FALSE
Search result spec prec mz: 527.5783 Actual spec prec mz: 527.57827
Fragments matched per AA: 2.38 Proportion of top 20 peaks matched: 0.55
Intensity
M/Z
B
Scatterplot of predicted elution time
Fitting R2: 0.702
Novel peptide residual Z score: 0.666
Number of peptides: 1315
Pred. hydrophobicity
Experimental scan number
C
Distributions of residuals from best−fit line
of predicted RT vs Expt. scan number
Line: Z score of novel peptide
Z: 0.666
count
Z scores of residuals from best linear fit
